# Effects of increasing dietary arginine supply during the three first weeks after weaning on pig growth performance, plasma amino acid concentrations, and health status

**DOI:** 10.1093/tas/txae047

**Published:** 2024-04-01

**Authors:** Jorge Y Perez-Palencia, Christian D Ramirez-Camba, Keith Haydon, Kristine L Urschel, Crystal L Levesque

**Affiliations:** Department of Animal Science, South Dakota State University, Brookings, SD 57007, USA; Department of Animal Science, South Dakota State University, Brookings, SD 57007, USA; Department of Animal Science, University of Minnesota, St. Paul, MN 57008, USA; CJ Bio America Inc, Downers Grove, IL 60515, USA; Department of Animal and Food Sciences, University of Kentucky, Lexington, KY 40546, USA; Department of Animal Science, South Dakota State University, Brookings, SD 57007, USA

**Keywords:** functional amino acids, intestinal health, marketing weight, nursery diet, postweaning diarrhea, weaned pigs

## Abstract

A total of 425 weaned pigs (Exp. 1: 225 pigs [5.8 ± 0.9 kg]; Exp. 2: 200 pigs [6.1 ± 1.2 kg]) were used to determine the optimal dietary standardized ileal digestible (**SID**) arginine (**Arg**) level in early nursery diets based on growth and health responses. The basal diet in Exp.1 was formulated to meet SID Arg recommendation (0.66%; NRC, 2012) and in Exp. 2, SID Arg was set to simulate current industry practices for feeding nursery pigs (1.15 %). Basal diets were supplemented with 0.3%, 0.6%, 0.9%, and 1.2% of l-arginine to provide five levels of dietary SID Arg. Experimental diets were fed during phases I (days 0 to 7) and II (days 8 to 21) with common diets until market. Feed disappearance and body weight (**BW**) were measured on days 7, 14, 21, and 43. Final BW was recorded at first removal of pigs for market. Pen fecal score was assigned daily from days 0 to 21. Plasma immunoglobulin A (**IgA**) was determined on days 0, 7, and 14 and amino acids (**AAs**) concentration and plasma urea nitrogen (**PUN**) on days 0 and 14. Orthogonal polynomial contrasts were used to determine the linear and quadratic effects of dietary Arg. Optimal SID Arg was determined by fitting the data with piecewise regression, using growth performance as the primary response variable. In Exp. 1, dietary Arg linearly increased (*P* < 0.1) BW, average daily gain (**ADG**), and gain to feed ratio (**G:F**) ratio on day 21, as well as reduced (*χ*^2^ = 0.004) the percentage of pigs that lost weight (**PLW**) in week 1 by 29%. Dietary Arg resulted in linear improvement (*P* = 0.082) of ADG for the overall nursery period and quadratic improvement (*P* < 0.1) of final BW at marketing. In Exp. 2, dietary Arg linearly increased (*P* < 0.05) ADG and average daily feed intake (**ADFI**) in week 1, BW and ADFI (*P* < 0.1) on day 14, as well as reduced (*χ*^2^ ≤ 0.001) PLW in week 1. From days 0 to 21, G:F was improved quadratically (*P* < 0.1). Dietary Arg linearly increased (*P* < 0.1) ADG and BW on day 43. Dietary Arg supplementation decreased the incidence (*χ*^2^ < 0.05) of soft and watery feces during the first weeks after weaning and lower concentration of plasma IgA on days 7 and 14. Dietary Arg linearly and/or quadratically influenced plasma AA concentrations (*P* < 0.05), including an increase in Arg, Leu, Phe, Val, citrulline, ornithine, and PUN concentrations. Overall, weaned pigs exhibit optimal nursery growth performance and health when provided with dietary SID Arg ranging from 1.5% to 1.9%. This dietary range contributes to a reduction in the occurrence of fall-back pigs and improvements in final BW at marketing.

## INTRODUCTION

In swine production, the immediate postweaning period is commonly accompanied by intestinal disturbances resulting in increased diarrhea incidence, poor growth performance, greater susceptibility to diseases, and increased mortality, which in turn causes economic losses for swine producers (McLamb et al., 2013; Xiong et al., 2019). To overcome these challenges, weaned pig feed was commonly supplemented with antibiotics or pharmacological doses of zinc oxide (**ZnO**) and copper to control postweaning diarrhea and promote growth performance (Verstegen and Williams, 2002). However, the use of antibiotics for growth promotion in animal feed has been banned due to an increase in the number of antibiotic-resistant pathogens and the potential implications for human health (Marshall and Levy, 2011; FDA, 2017). Furthermore, high doses of ZnO in pig diets are a current environmental concern due to zinc excretion (Shurson et al., 2022). These concerns have generated greater interest in strategies to improve postweaning pig growth and health that reduce reliance on ZnO and antibiotics in nursery diets (Heo et al., 2013; Cheng et al., 2014; Oh et al., 2020; Pejsak et al., 2023).

Amino acids (**AAs**) are essential for intestinal development and function, especially those directly related with gut physiology (i.e., functional AAs, Wu et al., 2007). Within functional AAs, arginine (**Arg**) participates in important regulatory functions associated with nutrient metabolism, intestinal repair, cell proliferation, and immune response that contribute to the maintenance of a functional intestinal tract (Rhoads et al., 2004; Zhan et al., 2008; Wu et al., 2010a; Zheng et al., 2017). Under stressful or challenging conditions, such as weaning, the need for this AA may increase, making endogenous synthesis and even dietary intake insufficient to meet the demand, necessitating dietary supplementation (Chalvon-Demersay et al., 2021). Dietary supplementation of 0.6% to 1% Arg in young pig diets has been associated with improved growth performance (Hernandez et al., 2009; Wu et al., 2010b; Yun et al; 2020), enhanced intestinal development and morphology (Zhan et al., 2008; Wu et al., 2010b; Yao et al., 2011; Zheng et al., 2017), and reduced inflammatory responses (Yao et al., 2011; Zheng et al., 2017). Furthermore, under challenging conditions (deoxynivalenol-contaminated diet at 6 mg/kg), dietary supplementation with Arg significantly enhanced AA utilization and decreased circulating pro-inflammatory cytokines (Wu et al., 2013, 2015). However, depending on the balance relative to Lys, the excess Arg (1.94% to 3.27% total Arg and 1.63% total Arg in combination with 1.03% lysine) can result in adverse effects on pigs’ growth performance (Southern and Baker, 1982; Hagemeier et al., 1983).

Collectively, dietary Arg supplementation has been shown to improve intestinal health and, in some cases, the performance of weaned pigs during the postweaned period. However, current practical diet formulation for weaned pigs do not consider the critical regulatory roles of Arg related to pig gut health and functions, in part because dietary Arg content is typically above current dietary recommendations for the nursery phase (0.56 to 0.68 standardized ileal digestible (**SID**) Arg; NRC, 2012). Furthermore, the effects of increasing dietary Arg supply during the nursery period on pig performance up to market weight have not been studied. Thus, there is a need to define practical Arg requirements for weaned pigs and determine whether supplementation of synthetic Arg is needed for optimal pig growth and health within current industry formulations for nursery diets. We hypothesize that weaned pigs require more dietary Arg (above NRC recommendations and current industry formulation) for optimal health and growth performance after weaning. Therefore, the overall objective of this study was to determine the optimal dietary SID Arg in early nursery diets based on the growth and health responses of weaned pigs.

## MATERIAL AND METHODS

The experimental protocols used in this study were approved by the South Dakota State University Institutional Animal Care and Use Committee (IACUC #2108-045E). The experiments were conducted in the wean-to-finish barn at the South Dakota State University Swine Education and Research Facility, in Brookings, SD, USA. Each room contained 49 pens with a capacity for six pigs per pen until market (1 m^2^/pig). All pens contained one dry self-feeder and one-nipple waterer to allow ad libitum access to feed and water. The facility operated on mechanical ventilation, with the temperatures set at 28, 26, 24, 23, 22, and 22 °C for weeks 1 to 6 of the nursery period. For the grow-finish period, pigs remained in the same facility, with the temperatures set from 20 to 15 °C.

### Animals and Experimental Design

#### Experiment 1.

A total of 225 weaned pigs (5.8 ± 0.9 kg; 21 days), barrows and gilts, were used in a randomized complete block design. Pigs were weaned into 45 pens and assigned to one of five dietary treatments at weaning, each treatment with nine pens, five pigs per pen, and sex ratios maintained within body weight (**BW**) blocks. Diets were formulated to meet or exceed weaned pig’s nutrient recommendations (NRC, 2012) and provided in meal form in a three-phase nursery feeding program: phases I (days 0 to 7), II (days 8 to 21), and III (days 22 to 43). Basal diets were formulated to meet current dietary Arg recommendations (phase I: SID Arg 0.68%; phase II: SID Arg 0.61%) according to NRC (2012). To formulate these basal diets, soybean meal (**SBM**) was used in lower inclusion rates and synthetic AAs were included to meet AA recommendations (Table 1). l-Arginine was supplemented to the basal diets at 0%, 0.3%, 0.6%, 0.9%, and 1.2% to create a total of five dietary treatments with an average of 0.66%, 0.96%, 1.26%, 1.56%, and 1.86% SID Arg (analyzed values). Pigs were fed experimental diets during phases I and II of the nursery feeding program, then during phase III, all pigs received a common diet. To assess the effects of dietary treatments applied during the nursery period on subsequent growth performance, pigs were followed until marketing (first removal of pigs for market). Following the nursery period, all pigs were provided with the five-phase grow-finish feeding program used at the swine facility (Supplementary Table 1).

**Table 1.** Experimental diets (as-fed basis)^1^

Feeds	Experiment 1		Experiment 2		Phase III
	Phase I	Phase II	Phase I	Phase II	
Corn	53.28	63.25	48.94	53.63	62.35
Soybean meal	9.00	8.50	25.00	30.00	32.00
Dried Whey	25.00	18.00	15.00	8.00	0.00
HP300^2^	5.00	3.00	5.00	3.00	0.00
l-Lysine HCl	1.03	0.98	0.60	0.38	0.34
dl-Methionine	0.39	0.34	0.27	0.18	0.14
l-Threonine	0.39	0.37	0.24	0.16	0.16
l-Tryptophan	0.09	0.09	0.01	0.00	0.00
l-Valine	0.34	0.32	0.11	0.00	0.00
l-Isoleucine	0.20	0.20	0.00	0.00	0.00
l-Leucine	0.32	0.26	0.00	0.00	0.00
l-Phenylalanine	0.31	0.27	0.02	0.00	0.00
l-Histidine	0.20	0.17	0.05	0.00	0.00
Soybean oil	1.00	0.90	1.00	1.00	1.78
Monocalcium phosphate	1.05	1.04	1.15	1.02	0.90
Limestone	1.30	1.28	1.28	1.28	1.20
Salt	0.35	0.40	0.58	0.60	0.60
Vitamin premix^3^	0.05	0.05	0.05	0.05	0.05
Mineral premix^4^	0.15	0.15	0.15	0.15	0.15
Zinc oxide	0.42	0.30	0.42	0.42	0.20
Swine larvicide	0.13	0.13	0.13	0.13	0.13
*Calculated composition*					
ME, kcal/kg	3,430	3,413	3,469	3,476	3,350
CP, %	16.31	15.91	20.90	21.48	19.46
Lactose, %	18.22	13.12	10.93	5.83	0.00
SID Lys, %	1.50	1.35	1.50	1.35	1.23
SID Thr, %	0.88	0.79	0.88	0.79	0.73
SID Met, %	0.59	0.53	0.54	0.46	0.43
SID Met + Cys, %	0.82	0.74	0.82	0.74	0.68
SID Trp, %	0.25	0.22	0.25	0.24	0.20
SID Ile, %	0.77	0.69	0.82	0.84	0.72
SID Val, %	0.95	0.86	0.95	0.86	0.77
SID Arg, %	0.69	0.61	1.12	1.19	1.01
SID His, %	0.52	0.46	0.52	0.49	0.44
SID Leu, %	1.50	1.35	1.53	1.57	1.44
SID Phe, %	0.88	0.79	0.88	0.91	0.79
Calcium, %	0.85	0.80	0.85	0.80	0.70
ATTD P, %	0.41	0.36	0.41	0.36	0.30
STTD P, %	0.45	0.41	0.46	0.40	0.34

^1^ l-Arginine was included at 0%, 0.3%, 0.6%, 0.9%, and 1.2% in phases I and II to create a total of five dietary treatments in each experiment. CJ Bio America INC, Downers Grove, IL. Phases I (days 0 to 7), II (days 7 to 21), and 3 (days 21 to 43; common diet). SID, standard ileal digestibility; ATTD, apparent total tract digestibility; P, phosphorus; STTD, standardized total tract digestibility. Phase III diet was common in Exp. 1 and 2.

^2^Enzymatic treated soybean meal, HAMLET PROTEIN Inc., Findlay, OH, USA.

^3^J & R Distributing Inc., Lake Norden, SD, USA. Minimum provided per kilogram of diet: calcium 55 mg, vitamin A 11,000 IU, vitamin D3 1,650 IU, vitamin E 55 IU; vitamin B12 0.044 mg, menadione 4.4 mg, biotin 0.165 mg, folic Acid 1.1 mg, niacin 55 mg, d-pantothenic acid 60.5 mg, vitamin B16 3.3 mg, riboflavin mg, 9.9 thiamin 3.3 mg.

^4^J & R Distributing. Minimum provided per kilogram of diet: copper 16.5 ppm, manganese 44.1 ppm, selenium 0.03 ppm, zinc 165 ppm.

#### Experiment 2.

A total of 200 weaned pigs (6.1 ± 1.2 kg; 21 days), barrows and gilts, were used in a randomized complete block design. Pigs were weaned into 40 pens and assigned to one of five dietary treatments at weaning, each treatment with eight pens, five pigs per pen, and sex ratios maintained within BW blocks. Experimental diets were formulated to meet or exceed weaned pig’s nutrient recommendations (NRC, 2012) and provided in meal form in a three-phase nursery feeding program similar to Exp 1. In order to increase dietary challenge/stress during the first weeks after weaning and simulate current industry practices, basal diets were formulated with higher inclusion of SBM and lower inclusion of whey (less-complex diet, Collins et al., 2017; Koo et al., 2017) more reflective of current commercial nursery diets. As a result, basal diets contained 1.12% and 1.19% of SID Arg for phases I and II, respectively. l-Arginine was supplemented to the basal diet at 0%, 0.3%, 0.6%, 0.9%, and 1.2% to create a total of five dietary treatments with an average of 1.15%, 1.45%, 1.75%, 2.05%, and 2.35% SID Arg. Pigs were fed experimental diets during phases I and II of the nursery feeding program, then during phase III, all pigs received a common diet. Similar to Exp. 1, pigs were followed until marketing (first removal of pigs for market) and provided with the standard grow-finish feeding program at the swine facility.

Following diet mixing, feed samples were collected for each nursery phase in both studies. As feed was transferred from the mixer, feed samples were taken at intervals to complete a 1-kg sample. Feed samples were stored at 4 °C until analysis.

### Experimental Procedures

All experimental procedures were the same for both studies. To increase weaning-associated stress and mimic commercial weaning practices, at weaning, all pigs in each experiment were loaded onto a trailer and transported in the same route for 3 h prior to arriving at the wean-to-finish facility (mostly highway driving). For Exp. 1, weaning was conducted in September of 2021 while for Exp. 2 weaning was conducted in October of 2021. Daily animal care observations included pig behavior, recording daily room temperature (high and low temperature), checking waterers and feeders, and pharmaceutical treatment of pigs if needed. Pigs were treated when exhibiting clinical signs of illness and the treatment dose, product used, date given, pig and pen identification, and reason administered were recorded throughout the experimental period. Pig mortality/removal was recorded during the experiment, including day, time, pig and pen identification, BW, and reason for death/removal.

### Growth Performance

Pigs were weighed on days 0, 7, 14, 21, and 43 of the nursery period. Feed disappearance was measured simultaneously with BW. Average daily gain (**ADG**), average daily feed intake (**ADFI**), and gain:feed ratio (**G:F**) were calculated based on pigs’ BW and feed intake. One day prior to the first cut of pigs for marketing, final individual pig weights were recorded and data collection stopped.

### Health Measurements and Plasma-Free AA Concentration

Fecal scoring was assessed daily from days 0 to 21 and three times a week from days 21 to 43. Fecal consistency, on a pen basis was used to assign pen fecal score (Pedersen and Toft, 2011). The four consistency categories were: score 1 = firm and shaped, score 2 = soft and shaped, score 3 = loose, and score 4 = watery, where scores of 1 and 2 represented normal feces and scores of 3 and 4 represented diarrhea. For each pen, a single trained observer assigned the relative proportion of visible feces within each category for all pens, as well as an overall pen score (Perez-Palencia et al., 2021).

On days 7 and 14, blood sample was collected from one pig/pen (same pig within the average BW of the pen based on day 7 BW) in fed state using 6-mL sodium heparin BD Vacutainers (Becton, Dickinson and Company, Franklin Lakes, NJ, USA). In addition, the day before weaning, 12 average pigs were randomly selected for blood collection (named as day 0 collection). Plasma immunoglobulin A (**IgA**) was determined on days 0, 7, and 14 and AA concentration and plasma urea nitrogen (**PUN**) were determined on days 0 and 14. Plasma was collected by centrifugation (3,000 × *g*, 15 min, 4 °C), allocated into 1.5-mL microcentrifuge tubes, and stored at −20°C until analysis (CR412, Jouan Inc., Winchester, VA, USA).

### Chemical Analysis

Nutrient analyses of feed samples were conducted at the Agricultural Experiment Chemical Laboratories, University of Missouri (Columbia, MO). Feed samples were analyzed for dry matter (**DM**; AOAC Official Method 934.01, 2006), crude protein (**CP**; AOAC Official Method 990.03, 2006), and a complete AA profile (AOAC Official Method 982.30 E (a,b,c)).

The total concentration of IgA in the plasma of pigs was measured according to the method described by Chaytor et al. (2011) using commercially available ELISA kits (Bethyl Laboratories, Inc., Montgomery, TX). Each sample was analyzed in duplicate. The optical density (**OD**) value was read at 450 nm within 30 min by an ELISA plate reader (SpectraMAX190, Molecular Devices, Sunnyvale, CA, USA). A standard curve of OD value versus IgA concentration was generated and the serum IgA concentration was then determined according to the standard curve.

Plasma-free AA concentrations were measured using reverse phase HPLC (3.9 × 300 mm PICO-TAG reverse phase column; Waters) of phenylisothiocyanate derivatives as previously described (Urschel et al., 2011). Plasma PUN was measured using the Alfa Wassermann Clinical Chemistry System (Alfa Wassermann Diagnostic Technologies, LLC, West Caldwell, NJ, USA).

### Statistical Analysis

The UNIVARIATE procedure of SAS was used to confirm the homogeneity of variance and to analyze for outliers. Orthogonal polynomial contrasts were used to determine the linear and quadratic effects of increasing dietary levels of Arg. Pen was used as the experimental unit and BW as the blocking factor. In addition, optimal SID Arg to improve weaned pigs’ performance and final BW was determined by fitting the data with piecewise regression, specifically using the linear plateau and two-piece segmented regression. For fecal scores analysis and percentage of pigs that lost weight during the first week postweaning, data were analyzed using the PROC FREQ procedure in SAS. For all statistical tests, significant differences were reported at *P* < 0.05 and tendencies for significance at 0.05 ≤ *P* ≤ 0.10.

## RESULTS

The analyzed chemical composition of experimental diets used in the present study corresponded to the targets in the diet formulations and were within the tolerance of normal variance (Supplementary Table 2). Particularly, Arg-to-Lys ratio increased as expected across dietary treatments.

### Growth Performance

In Exp. 1, increasing dietary Arg supply linearly increased (*P* < 0.100) ADG during the first 2 wk after weaning, as well as BW on days 14 and 21 (Table 2). In comparison to the basal diet, in week 1, the percentage of pigs that lost weight in pens fed diets containing 1.26% and 1.56% SID Arg was reduced (*χ*^2^ = 0.004) by 29%. Considering the three first weeks postweaning, increasing dietary Arg supply linearly improved (*P* < 0.050) ADG and G:F ratio. From days 21 to 43, there were no differences between dietary treatments for growth performance. Over the entire nursery period, increasing dietary arginine supply during the three first weeks after weaning resulted in linear improvement (*P* = 0.082) of ADG. A quadratic response (*P* < 0.100) for final BW at marketing as a result of increasing Arg supply during the nursery period was observed, where 1.56% SID Arg promoted the greatest final BW (4.5% increase when compared with the basal diet).

**Table 2.** Effects of increasing dietary arginine supply during the three first weeks after weaning on pig growth performance during nursery period and final market weight (Exp. 1)^1^

Item	SID Arg, %					SEM	*P*-value	
	0.66	0.96	1.26	1.56	1.86		Linear	Quadratic
BW day 0, kg	5.79	5.79	5.81	5.79	5.79	0.039	0.939	0.745
*Period, days 0 to 7*								
BW day 7, kg	6.20	6.21	6.37	6.27	6.28	0.078	0.276	0.245
ADG, g	57.78	61.79	78.97	75.30	71.81	10.025	0.095	0.242
ADFI, g	95.48	108.33	116.85	110.17	113.47	8.308	0.166	0.174
G:F	0.58	0.55	0.66	0.70	0.62	0.059	0.139	0.388
PLW^2^, %	15.56	13.33	11.11	11.11	13.20	*χ* ^2^ = 0.004		
*Period, days 7 to 14*								
BW day 14, kg	7.39	7.41	7.62	7.65	7.53	0.102	0.048	0.099
ADG, g	166.16	171.32	178.55	197.96	179.28	10.417	0.071	0.291
ADFI, g	280.24	263.77	289.86	288.74	284.67	11.684	0.296	0.859
G:F	0.60	0.63	0.61	0.69	0.63	0.030	0.151	0.309
*Period, days 14 to 21*								
BW day 21, kg	9.66	9.69	10.02	10.23	9.97	0.202	0.061	0.427
ADG, g	328.05	325.88	342.88	368.09	347.92	19.607	0.208	0.824
ADFI, g	526.56	498.59	527.99	555.09	535.33	23.124	0.141	0.116
G:F	0.62	0.65	0.65	0.65	0.66	0.647	0.235	0.227
*Period, days 0 to 21*								
ADG, g	184.21	185.71	200.14	211.21	199.71	9.501	0.043	0.337
ADFI, g	300.90	290.37	311.49	316.36	311.24	11.641	0.142	0.806
G:F	0.61	0.63	0.64	0.67	0.64	0.013	0.045	0.137
*Period, days 21 to 43*								
BW day 43, kg	20.54	20.46	21.04	21.70	21.00	0.574	0.147	0.498
ADG, g	487.58	486.74	501.00	521.06	501.66	20.597	0.249	0.586
ADFI, g	826.21	812.35	850.32	855.95	846.34	38.500	0.400	0.769
G:F	0.60	0.60	0.60	0.60	0.61	0.595	0.780	0.638
*Period, days 0 to 43*								
ADG, g	338.74	340.31	354.10	369.10	354.24	13.788	0.082	0.438
ADFI, g	563.42	551.22	581.01	587.79	578.68	23.103	0.270	0.746
G:F	0.61	0.62	0.62	0.63	0.61	0.015	0.662	0.470
Final BW^3^	112.03	112.91	115.31	116.85	113.12	1.986	0.261	0.098

^1^ l-Arginine was included at 0%, 0.3%, 0.6%, 0.9%, and 1.2% in phases I and II to create a total of five dietary treatments in each experiment. Phases I (days 0 to 7), II (days 7 to 21), and 3 (days 21 to 43; common diet).

^2^PLW: percentage of pigs that lost weight during the first week postweaning.

^3^Final BW was determined 1 day prior to the first marketing event of the group.

In Exp. 2, increasing dietary Arg supply linearly increased (*P* < 0.050) ADG and ADFI during the first week after weaning, as well as BW and ADFI (*P* < 0.100) on day 14 (Table 3). In comparison to the basal diet, in week 1. the percentage of pigs that lost weight in pens fed diets containing 1.45%, 1.75%, and 2.05% SID Arg was reduced (*χ*^2^ ≤ 0.001) by 80%. From days 0 to 21, a quadratic response (*P* < 0.100) for G:F as a result of increasing Arg supply was observed, where 1.45% and 1.75% SID Arg promoted the greatest response. From days 21 to 43, ADG and ADFI linearly increased (*P* < 0.100) with Arg supplementation. Over the entire nursery period, increasing dietary Arg supply during the three first weeks after weaning resulted in linear improvement (*P* < 0.100) of ADG and BW on day 43. No statistical differences (*P* > 0.050) were detected at marketing; however, supplying 1.75% SID Arg or above numerically increased final BW by approximately 4 kg.

**Table 3.** Effects of increasing dietary arginine supply during the three first weeks postweaning on pig growth performance during nursery period and final market weight (Exp. 2)^1^

Item	SID Arg, %					SEM	*P*-value	
	1.15	1.45	1.75	2.05	2.35		Linear	Quadratic
BW day 0, kg	6.10	6.11	6.11	6.11	6.10	0.049	0.990	0.852
*Period, days 0 to 7*								
BW day 7, kg	6.53	6.57	6.63	6.70	6.63	0.103	0.325	0.479
ADG, g	61.41	69.14	78.49	89.77	85.01	11.691	0.048	0.604
ADFI, g	108.07	108.32	112.47	126.77	124.46	7.663	0.044	0.862
G:F	0.57	0.62	0.70	0.69	0.67	0.057	0.165	0.306
PLW^2^, %	22.50	3.67	5.00	5.00	10.00	*χ* ^2^ ≤ 0.001		
*Period, days 7 to 14*								
BW day 14, kg	7.56	7.44	7.94	8.01	7.79	0.145	0.097	0.924
ADG, g	175.02	160.77	182.36	180.39	177.83	12.410	0.558	0.997
ADFI, g	280.25	262.53	267.63	300.60	286.94	10.270	0.052	0.182
G:F	0.60	0.66	0.68	0.59	0.62	0.040	0.784	0.301
*Period, days 14 to 21*								
BW day 21, kg	9.79	9.21	10.04	10.02	9.86	0.244	0.264	0.980
ADG, g	286.69	283.19	292.70	291.74	283.26	19.714	0.980	0.797
ADFI, g	473.27	445.65	490.32	484.41	489.83	16.562	0.214	0.815
G:F	0.61	0.62	0.60	0.60	0.58	0.030	0.465	0.759
*Period, days 0 to 21*								
ADG, g	178.11	178.47	186.91	186.40	178.82	9.436	0.562	0.697
ADFI, g	285.13	282.74	290.14	303.93	300.41	9.173	0.132	0.515
G:F	0.61	0.64	0.64	0.61	0.59	0.018	0.295	0.098
*Period, days 21 to 43*								
BW day 43, kg	19.41	18.93	20.24	20.99	20.74	0.600	0.081	0.707
ADG, g	460.19	446.16	485.72	522.20	517.54	22.283	0.058	0.536
ADFI, g	760.45	743.28	791.71	850.63	855.36	37.454	0.055	0.533
G:F	0.61	0.60	0.61	0.61	0.61	0.023	0.880	0.921
*Period, days 0 to 43*								
ADG, g	301.70	295.45	328.49	346.06	340.29	13.714	0.072	0.645
ADFI, g	542.55	548.40	540.92	577.28	577.89	31.015	0.357	0.789
G:F	0.59	0.56	0.61	0.60	0.59	0.020	0.601	0.897
Final BW^3^	104.45	103.02	107.94	108.69	108.78	2.888	0.412	0.572

^1^ l-Arginine was included at 0%, 0.3%, 0.6%, 0.9%, and 1.2% in phases I and II to create a total of five dietary treatments in each experiment. Phases I (days 0 to 7), II (days 7 to 21), and 3 (days 21 to 43; common diet).

^2^PLW: percentage of pigs that lost weight during the first week postweaning.

^3^Final BW was determined 1 day prior to the first marketing event of the group.

### Postweaning Health Status

**Figure 1.**
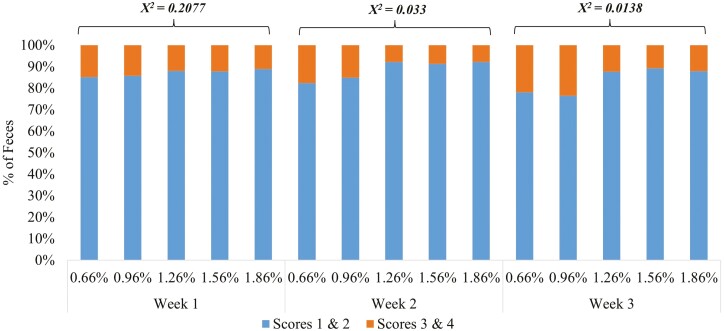
Effects of increasing dietary arginine supply during the three first weeks postweaning on fecal consistency of weaned pigs (Experiment 1). l-Arginine was included at 0%, 0.3%, 0.6%, 0.9%, and 1.2% in phases I and II to create a total of five dietary treatments. Phases I (days 0 to 7), II (days 7 to 21), and 3 (days 21 to 43; common diet). Fecal score: scores 1 and 2 represented normal feces and scores of 3 and 4 represented diarrhea.

**Figure 2.**
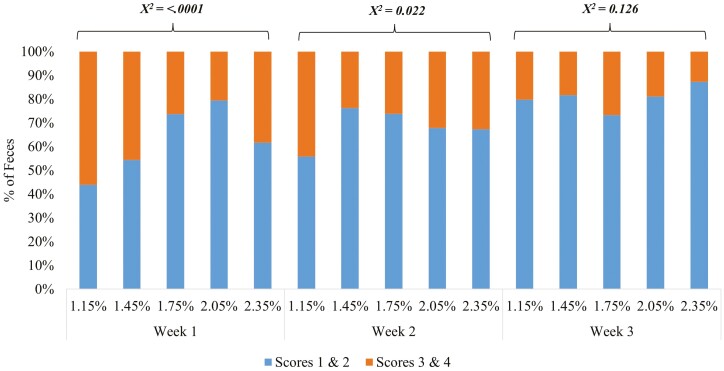
Effects of increasing dietary arginine supply during the three first weeks postweaning on fecal consistency of weaned pigs (Experiment 2). l-Arginine was included at 0%, 0.3%, 0.6%, 0.9%, and 1.2% in phases I and II to create a total of five dietary treatments. Phases I (days 0 to 7), II (days 7 to 21), and 3 (days 21 to 43; common diet). Fecal score: scores 1 and 2 represented normal feces and scores of 3 and 4 represented diarrhea.

During weeks 2 and 3 (Exp. 1), pigs fed diets containing 1.26% to 1.86% SID Arg had less incidence (*χ*^2^ < 0.05) of soft and watery feces (Fig. 1) and lower plasma IgA concentration on days 7 and 14 (*P* < 0.05). In Exp. 2, pigs fed diets containing increasing levels of SID Arg had less incidence (*χ*^2^ < 0.05) of soft and watery feces during the first 2 wk after weaning (Fig. 2). Plasma IgA concentration on day 7 was lower (*P* < 0.100) when 1.75% SID Arg was provided. When compared to preweaning state, plasma IgA concentrations were lower after weaning in both experiments, independent of Arg levels in the diet.

### Plasma AA Concentration and PUN

In Exp. 1, increasing dietary Arg supply linearly and/or quadratically influenced plasma AA concentrations (*P* < 0.05) except for His, Thr, Ala, Asp, and Tau (Table 4). Plasma concentration of Arg and PUN increased linearly while concentration of all other AA decreased linearly and/or quadratically. Preweaning Arg, Trp, Asn, Citr, Gly, Orn, Pro, Tyr, Ser, and PUN concentrations in plasma were higher when compared to postweaning concentrations while Met, Thr, Asp, and Glu were lower. In Exp. 2 (Table 5), increasing dietary Arg supply linearly and/or quadratically increased plasma Arg, Leu, Phe, Val, Citr, and PUN concentrations (*P* < 0.05). When compared to preweaning state, plasma Ile, Thr, Trp, Ala, Asp, Glu, Orn, and PUN concentrations were higher after weaning while His, Leu, Val, Citr, Pro, and Tyr concentrations were lower. Overall, plasma Arg concentration increased up to 2.05 SID Arg while most of other AA concentrations were influenced up to 1.56 SID Arg in Exp. 1 and 1.75 SID Arg in Exp. 2.

**Table 4.** Effects of increasing dietary arginine supply during the three first weeks postweaning on plasma AAs, IgA, and plasma urea nitrogen (Exp. 1)^1^

Item	Prewean^2^	SID Arg, %					SEM	*P*-value^3^		
		0.66	0.96	1.26	1.56	1.86		Linear	Quadratic	Contrast
IgA day 7, mg/mL	0.33	0.38	0.32	0.28	0.30	0.28	0.017	0.056	0.679	0.028
IgA day 14, mg/mL	0.39	0.44	0.40	0.30	0.24	0.31	0.012	<0.001	<0.001	0.009
PUN^4^ day 14, mg/dl	6.25	2.33	3.22	3.67	4.33	7.22	0.953	0.001	0.244	0.030
Arginine	121.36	50.52	50.58	65.87	111.49	140.81	13.667	<0.001	0.090	0.004
Histidine	280.56	333.20	273.91	381.75	287.28	307.21	47.286	0.798	0.806	0.404
Isoleucine	71.66	102.17	61.45	58.38	68.65	65.88	8.626	0.021	0.009	0.964
Leucine	147.84	181.05	132.91	125.83	135.23	129.89	12.315	0.014	0.033	0.551
Lysine	259.07	485.61	258.25	215.15	230.52	191.50	59.912	0.002	0.059	0.745
Methionine	54.83	103.95	70.14	69.15	63.64	61.89	8.408	0.002	0.065	0.015
Phenylalanine	64.72	89.63	67.17	67.44	68.81	69.96	5.138	0.026	0.016	0.124
Threonine	189.41	356.94	208.29	337.08	356.10	291.50	43.018	0.902	0.720	0.003
Tryptophan	6.54	4.86	2.92	3.11	4.36	3.00	0.600	0.302	0.264	<0.001
Valine	221.18	322.35	212.90	207.25	235.69	225.24	20.873	0.013	0.005	0.312
*Dispensable AA*										
Alanine	668.35	820.66	703.77	636.68	724.26	691.64	65.084	0.267	0.200	0.433
Asparagine	111.40	120.50	84.84	76.99	74.99	72.08	6.647	<0.001	0.007	0.001
Aspartate	64.57	138.15	126.39	118.98	129.38	122.20	10.419	0.386	0.493	<0.001
Citrulline	147.43	94.25	78.58	73.54	85.79	87.75	7.410	0.806	0.065	<0.001
Glutamate	493.81	570.99	501.78	407.29	656.53	478.62	60.484	0.876	0.587	0.600
Glutamine	343.18	531.85	455.92	387.76	368.83	370.61	44.437	0.007	0.234	0.050
Glycine	1,328.49	1,412.82	1,102.41	1,012.95	1,033.43	950.53	74.592	<0.001	0.050	0.003
Ornithine	239.60	89.54	71.92	83.27	132.18	154.10	12.679	<0.001	0.018	<0.001
Proline	468.46	237.68	194.01	188.24	198.41	193.04	13.644	0.056	0.078	<0.001
Taurine	37.34	88.69	94.59	27.32	86.46	75.54	39.231	0.783	0.531	0.278
Tyrosine	146.34	75.03	54.15	58.41	56.61	54.85	3.958	0.004	0.036	<0.001
Serine	270.18	191.51	131.77	140.33	135.48	125.98	9.622	<0.001	0.020	<0.001

^1^ l-Arginine was included at 0, 0.3, 0.6, 0.9, and 1.2% in phases I and II to create a total of five dietary treatments in each experiment. Phases I (days 0 to 7), II (days 7 to 21), and 3 (days 21 to 43; common diet).

^2^The day before weaning, 12 pigs were randomly selected for blood collection.

^3^Contrast: indicates a comparison between prewean and postwean plasma analysis.

^4^PUN, plasma urea nitrogen.

**Table 5.** Effects of increasing dietary arginine supply during the three first weeks postweaning on plasma AAs, IgA, and plasma urea nitrogen (Exp. 2)^1^

Item	Prewean^2^	SID Arg, %					SEM	*P*-value^3^		
		1.15	1.45	1.75	2.05	2.35		Linear	Quadratic	Contrast
IgA day 7, mg/mL	0.43	0.40	0.36	0.34	0.39	0.35	0.017	0.926	0.583	<0.001
IgA day 14, mg/mL	0.46	0.41	0.40	0.36	0.38	0.41	0.030	0.775	0.187	0.025
PUN day 14, mg/dl	6.92	8.25	10.25	13.75	13.38	17.13	2.152	0.004	0.963	0.003
Arginine	142.97	118.58	133.95	166.05	230.27	184.64	33.259	0.037	0.475	0.403
Histidine	58.16	35.32	36.44	46.72	41.48	42.92	4.112	0.129	0.340	<0.001
Isoleucine	70.35	89.49	99.82	100.04	95.78	81.64	7.110	0.386	0.052	0.002
Leucine	146.62	116.40	128.96	138.01	135.33	103.67	9.261	0.519	0.007	0.019
Lysine	212.98	275.23	253.51	244.45	302.92	188.28	42.634	0.366	0.466	0.307
Methionine	39.36	42.57	45.01	44.32	51.29	42.57	6.476	0.761	0.549	0.311
Phenylalanine	54.62	52.13	62.65	66.16	55.92	59.69	4.022	0.514	0.079	0.271
Threonine	137.53	171.39	162.22	190.85	173.25	185.53	26.999	0.648	0.974	0.095
Tryptophan	6.73	9.67	12.25	9.88	13.87	9.42	2.333	0.881	0.385	0.034
Valine	208.48	101.03	111.70	143.28	131.69	115.70	13.997	0.273	0.074	<0.001
*Dispensable AA*										
Alanine	534.75	659.92	666.44	649.37	763.66	595.83	56.795	0.864	0.314	0.008
Asparagine	100.47	113.60	126.25	113.00	116.31	108.93	10.549	0.567	0.555	0.103
Aspartate	95.35	164.17	160.45	151.55	152.88	134.96	10.378	0.052	0.643	<0.001
Citrulline	146.64	89.03	105.91	85.92	109.41	123.44	9.842	0.026	0.312	<0.001
Glutamate	412.82	570.97	629.74	590.33	524.78	518.65	87.230	0.294	0.506	0.007
Glutamine	340.35	319.16	385.72	327.80	337.27	300.75	19.778	0.181	0.069	0.724
Glycine	1,115.72	1,399.75	1,231.47	1,017.76	1,153.92	1,207.33	87.013	0.104	0.021	0.253
Ornithine	114.13	214.64	220.16	208.24	215.48	285.09	38.675	0.277	0.318	0.001
Proline	402.65	221.39	224.88	227.89	279.09	218.81	23.163	0.507	0.366	<0.001
Taurine	258.67	267.41	266.56	221.38	253.16	268.44	30.980	0.909	0.353	0.904
Tyrosine	135.13	80.16	95.30	86.01	81.88	74.60	7.792	0.326	0.182	<0.001
Serine	207.48	200.06	197.39	170.59	169.72	177.43	14.412	0.119	0.393	0.107

^1^ l-Arginine was included at 0%, 0.3%, 0.6%, 0.9%, and 1.2% in phases I and II to create a total of five dietary treatments in each experiment. Phases I (days 0 to 7), II (days 7 to 21), and 3 (days 21 to 43; common diet).

^2^The day before weaning, 12 pigs were randomly selected for blood collection.

^3^Contrast: indicates a comparison between prewean and postwean plasma analysis.

^4^PUN, plasma urea nitrogen.

### Breakpoint Analysis

**Figure 3.**
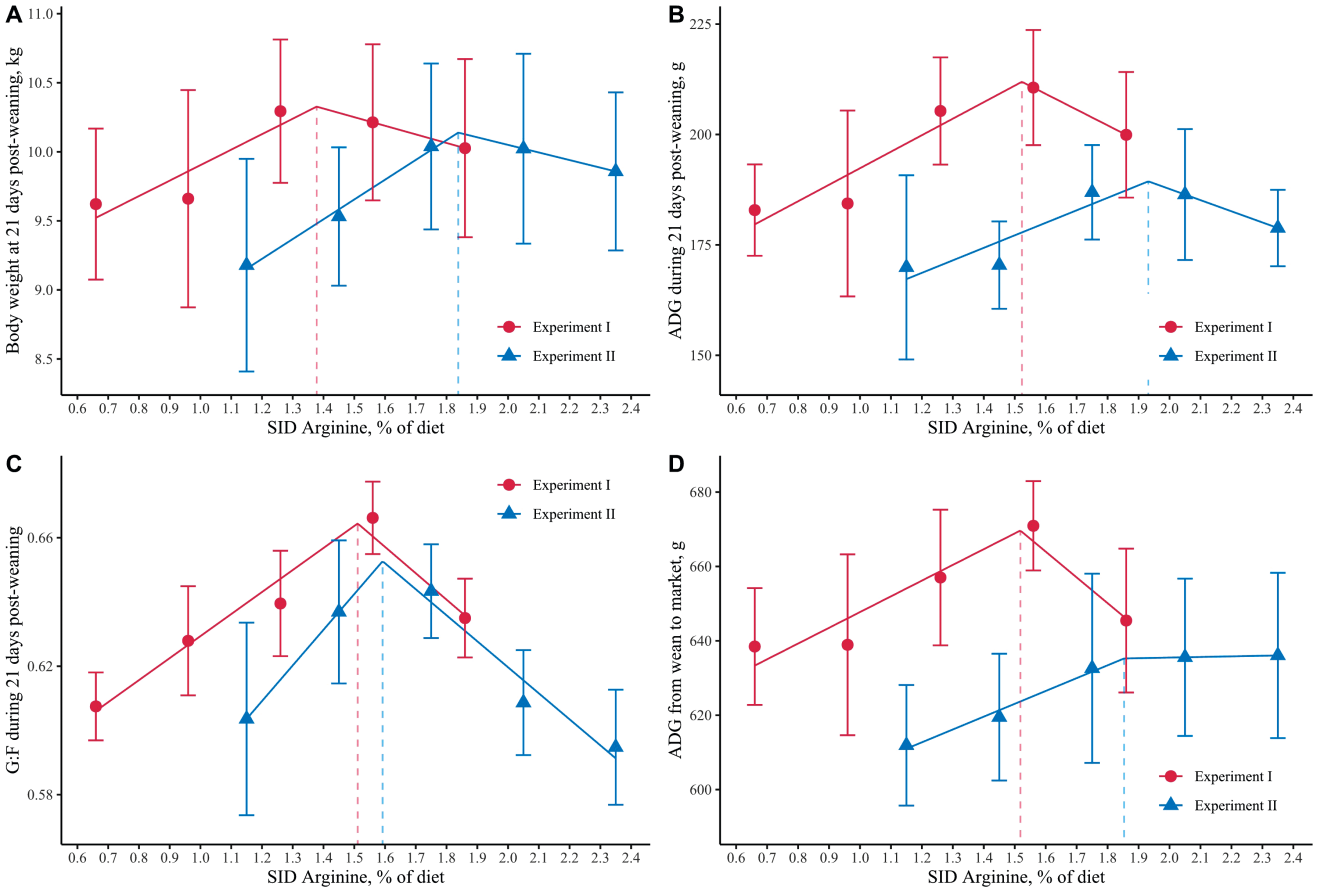
Optimal SID arginine for weaned pigs based on growth performance during the three first weeks postweaning and ADG until market weight. Weaned pigs require between 1.38% and 1.93% SID Arginine for optimal growth performance during the first weeks postweaning and ADG until market weight.

The segmented regression analysis revealed differences between Exp. 1 and 2 in breakpoint estimation for SID Arg intake. As depicted in Fig. 3A, the breakpoint based on BW on day 21 postweaning was 1.38% SID Arg for Exp. 1 and 1.83% SID Arg for Exp. 2. Similarly, based on ADG, Exp. 1 exhibited a breakpoint at 1.52% SID Arg, while in Exp. 2, it was 1.93% SID Arg (Fig. 3B). Examining G:F, the breakpoints for Exp. 1 and 2 were 1.51% and 1.59% SID Arg, respectively, as illustrated in Fig. 3C. Additionally, the inclusion of SID Arg up to 1.52% in Exp. 1 and up to 1.85% in Exp. 2 increased ADG by 36.3 and 24.3 g/d during the wean-to-market period (Fig. 3D). These differences in ADG correspond to improvements of 5.4 and 3.6 kg BW at marketing for Exp. 1 and 2, respectively. Taken together, the segmented analysis indicated that weaned pigs require between 1.38% and 1.93% SID Arginine during the first weeks postweaning, as determined by BW, ADG, and G:F.

## DISCUSSION

### Growth Performance

Increasing dietary Arg content up to 1.5% SID (or 4.7 g/d) Arg in Exp. 1 and up to 1.9% (or 5.7 g/d) SID Arg in Exp. 2 during the first 3 wk after weaning improved BW, ADG, and G:F on day 21. This improvement extended to the whole nursery period, the ADG over the entire wean-to-finish period, and resulted in an enhanced final BW at marketing. These results align with Wu et al. (2010b), who identified Arg deficiency in piglets during the initial days postweaning. Considering results from Exp. 1, where the basal diet met NRC (2012) requirements for SID Arg intake (1.8 to 2.9 g/d), increasing dietary Arg supply resulted in improved pig growth performance, which validates the hypothesis that weaned pigs require dietary Arg above NRC recommendations for optimal performance after weaning. Similarly, according to results from Exp. 2, increasing dietary Arg supply above current industry formulation for nursery diets resulted in benefits on pig performance, which suggests that the current diets formulated for weaned pigs may be Arg deficient.

Our results also suggest that Arg excesses may have a negative impact on pig growth performance or simply do not result in further benefits. Arginine and Lys are both basic AA and compete for the same transport system for intestinal absorption (Wu et al., 2004). Therefore, increasing dietary Arg might result in Lys deficiency consequently compromising performance (Zhan et al., 2008; Wu et al., 2016). In fact, previous literature on Arg supplementation has shown reduced performance in pigs when feeding excess levels of Arg (Southern and Baker, 1982; Hagemeier et al., 1983; Wu et al., 2010b). However, dietary supplementation of Arg up to 2% (or 630 mg/kg BW/day) did not have any adverse effect on weaned pigs and was considered as a safe limit (Hu et al., 2015). These observations align with the findings of the current study, as performance was not reduced within this range of Arg intakes when compared to the basal diets.

Our findings indicate that the Arg requirement varies depending on the composition of the diet. These variations in the calculated Arg requirement could be linked to differences in the quality of macronutrients present in the basal diet and consequently in the level of challenge that such diets present to pigs. In Exp. 1, the protein was on average 15% coming from animal sources, 15% from crystalline AA, and 70% from plant sources. In Exp. 2, the protein was on average 6% coming from animal sources, 4% from crystalline AA, and 90% from plant sources. Thus, the protein quality differed between the two experiments. This difference may have influenced the requirements of SID Arg, as diets containing a greater proportion of protein from plant sources resulted in increased SID Arg requirement. Considering that SBM was the main protein source in these diets, it seems that pigs in Exp. 2 had a higher nutritional challenge at weaning, which can be evidenced by a higher incidence of diarrhea scores in comparison with Exp. 1. The inclusion of SBM in young pig diets is limited due to relatively high levels of antinutritional factors (ANFs) and nonstarch polysaccharides. The presence of ANFs such as protease inhibitors, lectins, phytoestrogens, oligo-saccharides, and phytin interfere with the digestion and absorption of nutrients, which contribute to variations in the nutritional value of SBM (Liener, 1994; Pettersson and Pntoppidan, 2013). In addition, two major antigens in SBM (glycinin and β-conglycinin) can induce allergic reaction and have been associated with inflammatory response in pigs (Wang et al., 2023). As a result, it has been reported that ANFs in SBM can impair growth performance and compromise intestinal health of pigs, especially when included in young pig diets (Song et al., 2010; Wang et al., 2014). In this study, the Arg effect on improving fecal consistency was more evident in the higher challenge scenario (Exp. 2) since the improvement in the incidence of diarrhea was detected from week 1 compared to Exp. 1 (less challenge), where Arg benefit was not detected until week 2.

Other titration experiments analyzed in the context of our results also suggest that variations in animal response to SID Arg intake may be explained by differences in diet composition. For example, in a recent study by Greiner et al. (2023), pigs were fed three dietary Arg treatments (1.35%, 1.55%, and 1.75% SID Arg) during the first 20 days postweaning. Greiner et al. (2023) provided diets containing animal protein (fish meal, plasma protein, and dried whey), plant-based protein (SBM), crystalline AA, and lactose, making their diets more closely resemble those provided in Exp. 1. The optimal SID Arg levels for maximizing growth performance calculated by Greiner et al. (2023) at 1.55% are closer to the levels estimated from Exp. 1 at 1.5% SID Arg. In addition, Tan et al. (2009) administered six dietary treatments to weaned pigs, varying in arginine content from 0.78% to 1.38%. Their experimental diets consisted of animal protein, crystalline AAs, and lactose, excluding plant-based protein. The growth performance of the pigs increased linearly, reaching optimal levels at 1.18% Arg. The results of the current study and the observations made based on Greiner et al. (2023) and Tan et al. (2009) suggest that the SID Arg requirement is influenced by the quality/composition of the diet. It is important to acknowledge, however, that Exp. 1 and 2 were not conducted simultaneously, and potential confounders may exist related to the specific conditions of the experiments, such as environmental conditions or the initial health status of the herd. Nevertheless, our results still provide important insights, especially regarding the range of dietary SID Arg content and the magnitude of the effect, for guiding the development of future studies.

The benefits of increasing dietary Arg supply during the first weeks after weaning transcended until marketing. Different parameters in the early life of pigs (< 10 wk of age or end of nursery) have been correlated to growth performance in later stages of life or even carcass characteristics at slaughter (Alvarenga et al., 2013; Smit et al., 2013; Du et al., 2015; López-Vergé et al., 2018). In this regard, light BW at nursery exit was a better indicator for slow growth in subsequent phases compared with light BW at weaning or birth (He et al., 2016). In addition, ADG during the first weeks postweaning has been associated with pig growth performance until market, particularly final market BW and/or days to reach market (Tokach et al.,1992; Wolter and Ellis, 2001; Faccin et al., 2020). Consequently, management and nutritional interventions in early life that maximize those long-term indicators of growth performance and profitability represent a promissory opportunity to improve overall pig productivity (Collins et al., 2017; Blavi et al., 2021). In the present study, Arg supplementation during the first 3 wk after weaning, increased ADG and BW on day 21, which consequently increased BW (Exp. 2) and ADG (Exp. 1) over the entire nursery period and final BW at marketing. To our knowledge, this is one of the first studies reporting the effects of Arg supplementation during the nursery phase on growth performance parameters at marketing. This highlights the importance of assessing the impact of nutritional strategies in early stages of life on performance indicators until market in order to uncover their full growth potential.

### Postweaning Health Status and Circulatory AA Levels and PUN

Traditional methods for estimating AA requirements are based on the minimal levels of dietary AA required to maximize growth performance or protein retention, without considering other physiological responses (i.e., health status) (Hammond, 1944; NRC, 2012). However, growing evidence indicates that certain AA play important physiological functions related to nutrient metabolism, gene expression, cell proliferation, and immunity modulation (Wu, 2013; Le Floc’h et al., 2018; Tang et al., 2021; Luise et al., 2023). These functions become even more important when animals are under sanitary or stress challenges, such as weaning (Jayaraman and Nyachoti, 2017; Mou et al, 2019; Rodrigues et al., 2021).

The effects of dietary AA on maintaining or improving pig’s intestinal health around weaning are based on their roles as precursors of energy and functional molecules, as signaling molecules, and as microbiota modulators (Chalvon-Demersay et al., 2021; Liao 2021). In this regard, dietary Arg can positively contribute to gut health by supporting or restoring gut barrier function (Zhu et al., 2012) and improving gut immunological functions (Zheng et al., 2017). Arginine is converted into ornithine by the action of arginase and ornithine is used by ornithine decarboxylase to produce polyamines (putrescine, spermidine, and spermine), which are associated with intestinal mucosal protection and intestinal epithelial cell migration (Wang et al., 2009; Fritz, 2013). In addition, ornithine can be converted into proline by the action of ornithine aminotransferase. Proline is an important precursor in collagen synthesis and has been related with wound healing and cell migration in fibroblasts and epithelial cells (Luiking et al., 2012). In the current study, the plasma concentration of ornithine increased linearly and/or quadratically while proline levels tended to decrease as a result of increasing dietary Arg supply. On the other hand, arginine is a precursor of nitric oxide by the action of nitric oxide synthase (Wu, 2010). As reviewed by Fritz (2013), nitric oxide produced in inflammatory monocytes and dendritic cells can regulate inflammatory cytokine production, cell differentiation, and survival. Collectively, these mechanisms of action support the health indicator responses in this study: 1) improved intestinal integrity characterized indirectly by the reduction in plasma IgA concentrations; 2) reduced incidence of diarrhea; and 3) decreased fall-back pigs.

In this study, IgA was used as an indirect marker of intestinal integrity. Immunoglobulin A is produced by and secreted from mucosal tissues, especially in the intestine. Once in the intestinal lumen, along with mucus, IgA is considered the first line of defense against adhesion and invasion of pathogenic organisms and has been associated with the maintenance of the intestinal epithelial barrier function (Li et al., 2018; Pietro et al., 2019). When the intestinal barrier is compromised (increase permeability), IgA molecules that normally cannot be absorbed because of their size are translocated to the circulation, resulting in increased IgA concentration in the blood. Thus, decreased plasma IgA levels in the current study are suggestive of increased barrier integrity. Consequently, increasing levels of dietary Arg improved intestinal integrity after weaning. However, plasma IgA concentration as a measurement of intestinal permeability does not necessarily account for differences between intestinal IgA production and circulating (absorbed) IgA, which could be a limitation for this method (Vaerman et al., 1997).

Plasma concentrations of Arg and PUN increased linearly as expected, considering that l-arginine was supplemented to the basal diets. Some current methods for estimating AA requirements for pigs see these increases in plasma Arg and PUN as waste and potentially hazardous to the animal (France and Kebreab, 2008). However, these higher levels can be normal considering Arg metabolism to generate functional molecules that improve health functions. Indeed, there is accumulating evidence that AA ingestion at levels greater than those required for maximum growth performance (or maximum nitrogen retention) benefits a variety of physiological processes in the organism such as protein turnover (Young and Marchini, 1990) and transmethylation reactions (Robinson et al., 2016). As suggested by Ramirez-Camba and Levesque (2013), AA levels above those required for optimal growth are not necessarily detrimental or excessive to the animal but can actually improve its metabolic status, even more under stressful/challenging conditions.

## CONCLUSIONS

Weaned pigs exhibit optimal nursery growth performance and health when provided with dietary SID Arg ranging from 1.5% to 1.9%. This dietary range contributes to a reduction in the occurrence of fall-back pigs and results in increased final BW at marketing. The specific requirement appears to be influenced by the composition of the diet and/or level of nutritional challenge. Our findings indicate that, within current commercial practices, the optimal SID Arg requirement during the first 3 wk after weaning for optimizing pig performance throughout their entire productive lifespan is approximately 1.82%, which represents average daily intake of 5.6 and 2.7 g above NRC (2012) recommendations for phases I (week 1, 5 to 7 kg) and II (weeks 2 and 3, 7 to 11 kg), respectively. It is noteworthy that complex diets, including animal protein sources, crystalline AA, and/or lactose, have the potential to lower this requirement.

## Supplementary Material

txae047_suppl_Supplementary_Table_S1

txae047_suppl_Supplementary_Table_S2
