# Supplementary material for: Effects of increasing dietary arginine supply during the three first weeks after weaning on pig growth performance, plasma amino acid concentrations, and health status
Source: Transl Anim Sci. 2024 Apr 1;8:txae047. doi: 10.1093/tas/txae047 (PMC11034433; doi:10.1093/tas/txae047)
Supplement: txae047_suppl_Supplementary_Table_S1 [file txae047_suppl_supplementary_table_s1.docx]

**Supplementary Table 1.** SDSU standard 5-phase grow-finish feeding program (as-fed basis).

| Item | 5-phase grow-finish feeding program | | | | |
| --- | --- | --- | --- | --- | --- |
|  | 1 | 2 | 3 | 4 | 5 |
| BW, kg | 22 - 40 | 40 - 60 | 60 - 80 | 80 - 105 | 105 - 280 |
| Feed Budget, kg | 29.2 | 42.2 | 52.8 | 59.8 | 107.3 |
| Corn | 71.05 | 79.60 | 81.28 | 83.42 | 87.15 |
| Soybean meal | 26.21 | 18.00 | 16.51 | 14.66 | 11.01 |
| L-Lysine HCl | 0.36 | 0.30 | 0.22 | 0.21 | 0.20 |
| DL-Methionine | 0.15 | 0.00 | 0.00 | 0.00 | 0.00 |
| L- Threonine | 0.14 | 0.10 | 0.03 | 0.03 | 0.02 |
| L-Tryptophan | 0.01 | 0.00 | 0.00 | 0.00 | 0.00 |
| Limestone | 0.77 | 0.50 | 0.68 | 0.53 | 0.59 |
| Monocalcium phosphate | 0.31 | 0.50 | 0.28 | 0.14 | 0.03 |
| Salt | 0.40 | 0.40 | 0.40 | 0.40 | 0.40 |
| Mineral premix | 0.15 | 0.15 | 0.15 | 0.15 | 0.15 |
| Vitamin premix | 0.05 | 0.05 | 0.05 | 0.05 | 0.05 |
| Swine Toxin Binder | 0.25 | 0.25 | 0.25 | 0.25 | 0.25 |
| Swine Larvicide | 0.13 | 0.13 | 0.13 | 0.13 | 0.13 |
| Quantum Blue 5G Heat Stable | 0.02 | 0.02 | 0.02 | 0.02 | 0.02 |
| **Mixed feed** | **100.00** | **100.00** | **100.00** | **100.00** | **100.00** |
| **Calculated composition** |  |  |  |  |  |
| ME, kcal/kg | 3,300 | 3,300 | 3,300 | 3,320 | 3,325 |
| NE, kcal/kg | 2,483 | 2,519 | 2,519 | 2,551 | 2,571 |
|  |  |  |  |  |  |
| Calcium and Phosphorus |  |  |  |  |  |
| Available P | 0.26 | 0.28 | 0.28 | 0.21 | 0.18 |
| STTD P | 0.34 | 0.35 | 0.35 | 0.28 | 0.25 |
| STTD Ca | 0.44 | 0.43 | 0.43 | 0.33 | 0.32 |
|  | 1.29 | 1.22 | 1.23 | 1.18 | 1.28 |
| SID AA, % |  |  |  |  |  |
| Lysine | 1.10 | 0.84 | 0.84 | 0.70 | 0.60 |
| Methionine + Cystine | 0.68 | 0.47 | 0.49 | 0.44 | 0.41 |
| Threonine | 0.72 | 0.55 | 0.55 | 0.46 | 0.40 |
| Tryptophan | 0.20 | 0.15 | 0.15 | 0.13 | 0.11 |
| Isoleucine | 0.67 | 0.54 | 0.54 | 0.48 | 0.42 |
| Valine | 0.75 | 0.63 | 0.63 | 0.57 | 0.51 |
| Methionine | 0.41 | 0.23 | 0.25 | 0.21 | 0.20 |
| Cysteine | 0.27 | 0.24 | 0.24 | 0.22 | 0.21 |
| Leucine | 1.48 | 1.31 | 1.31 | 1.23 | 1.14 |
|  |  |  |  |  |  |
| Minerals |  |  |  |  |  |
| Sodium, % | 0.179 | 0.179 | 0.179 | 0.178 | 0.178 |
| Chloride, % | 0.238 | 0.238 | 0.238 | 0.237 | 0.236 |
| Magnesium, % | 0.172 | 0.159 | 0.159 | 0.151 | 0.147 |
| Potassium, % | 0.816 | 0.666 | 0.666 | 0.596 | 0.526 |
| Copper, ppm | 23 | 22 | 22 | 22 | 21 |
| Iodine, ppm | 0.36 | 0.36 | 0.36 | 0.36 | 0.36 |
| Iron, ppm | 231 | 222 | 223 | 213 | 213 |
| Manganese, ppm | 56 | 54 | 54 | 53 | 52 |
| Selenium, ppm | 0.42 | 0.40 | 0.40 | 0.40 | 0.39 |
| Zinc, ppm | 190 | 187 | 187 | 186 | 185 |
|  |  |  |  |  |  |
| Vitamins |  |  |  |  |  |
| A, IU/kg | 11000 | 11000 | 11000 | 11000 | 11000 |
| D, IU/kg | 1650 | 1650 | 1650 | 1650 | 1650 |
| E, IU/kg | 55 | 55 | 55 | 55 | 55 |
| Biotin, mg/kg | 0.28 | 0.27 | 0.26 | 0.26 | 0.25 |
| Choline, mg/kg | 1156 | 992 | 992 | 918 | 841 |
| Folic acid, mg/kg | 1.57 | 1.47 | 1.47 | 1.43 | 1.38 |
| Niacin, mg/kg | 78 | 78 | 78 | 78 | 78 |
| Pantothenic acid, mg/kg | 69 | 68 | 68 | 68 | 67 |
| Riboflavin, mg/kg | 12 | 11 | 11 | 11 | 11 |
| Thiamin, mg/kg | 6.63 | 6.66 | 6.66 | 6.69 | 6.70 |
| B6, mg/kg | 8.53 | 8.43 | 8.43 | 8.41 | 8.36 |
| B12, mg/kg | 4.41 | 4.41 | 4.41 | 4.41 | 4.41 |
