# Supplementary material for: Effects of increasing dietary arginine supply during the three first weeks after weaning on pig growth performance, plasma amino acid concentrations, and health status
Source: Transl Anim Sci. 2024 Apr 1;8:txae047. doi: 10.1093/tas/txae047 (PMC11034433; doi:10.1093/tas/txae047)
Supplement: txae047_suppl_Supplementary_Table_S2 [file txae047_suppl_supplementary_table_s2.docx]

**Supplementary Table 2.** Experimental diets (as-fed basis) – Analysed composition^1^

| **Item** | **Experiment 1** | | | | | | | | | | |  | **Experiment 2** | | | | | | | | | | |  | **Phase III** |
| --- | --- | --- | --- | --- | --- | --- | --- | --- | --- | --- | --- | --- | --- | --- | --- | --- | --- | --- | --- | --- | --- | --- | --- | --- | --- |
|  | **Phase I** | | | | |  | **Phase II** | | | | |  | **Phase I** | | | | |  | **Phase II** | | | | |  |  |
|  | **0.66** | **0.96** | **1.26** | **1.56** | **1.86** |  | **0.66** | **0.96** | **1.26** | **1.56** | **1.86** |  | **1.15** | **1.45** | **1.75** | **2.05** | **2.35** |  | **1.15** | **1.45** | **1.75** | **2.05** | **2.35** |  |  |
| DM, % | 90.80 | 90.96 | 91.02 | 91.00 | 90.96 |  | 90.32 | 90.70 | 90.44 | 90.48 | 90.47 |  | 91.14 | 91.06 | 90.80 | 91.11 | 90.91 |  | 89.70 | 89.81 | 89.94 | 89.92 | 90.08 |  | 89.24 |
| CP % | 16.28 | 17.03 | 17.40 | 18.03 | 18.43 |  | 14.99 | 15.14 | 15.13 | 16.44 | 17.24 |  | 19.90 | 21.31 | 22.05 | 22.86 | 22.85 |  | 19.26 | 20.98 | 22.19 | 22.63 | 23.01 |  | 18.81 |
| ***Total AA Composition, %*** | | | | | | | | | | | | | | | | | | | | | | | | | |
| Arg | 0.67 | 0.99 | 1.28 | 1.50 | 1.85 |  | 0.65 | 0.93 | 1.24 | 1.54 | 1.89 |  | 1.21 | 1.51 | 1.71 | 2.03 | 2.29 |  | 1.20 | 1.44 | 1.80 | 2.04 | 2.30 |  | 1.14 |
| Cys | 0.25 | 0.26 | 0.26 | 0.25 | 0.25 |  | 0.24 | 0.24 | 0.22 | 0.23 | 0.24 |  | 0.33 | 0.35 | 0.32 | 0.36 | 0.30 |  | 0.34 | 0.32 | 0.34 | 0.36 | 0.35 |  | 0.31 |
| His | 0.52 | 0.49 | 0.50 | 0.51 | 0.53 |  | 0.46 | 0.48 | 0.44 | 0.46 | 0.47 |  | 0.56 | 0.57 | 0.53 | 0.56 | 0.50 |  | 0.54 | 0.48 | 0.52 | 0.53 | 0.51 |  | 0.48 |
| Ile | 0.82 | 0.77 | 0.82 | 0.79 | 0.82 |  | 0.76 | 0.74 | 0.69 | 0.72 | 0.74 |  | 0.95 | 0.98 | 0.91 | 0.96 | 0.83 |  | 0.96 | 0.86 | 0.93 | 0.95 | 0.92 |  | 0.85 |
| Leu | 1.48 | 1.57 | 1.47 | 1.58 | 1.55 |  | 1.46 | 1.42 | 1.42 | 1.38 | 1.45 |  | 1.71 | 1.75 | 1.63 | 1.69 | 1.51 |  | 1.71 | 1.56 | 1.65 | 1.69 | 1.64 |  | 1.57 |
| Lys | 1.54 | 1.60 | 1.51 | 1.49 | 1.51 |  | 1.45 | 1.52 | 1.48 | 1.44 | 1.46 |  | 1.63 | 1.66 | 1.62 | 1.67 | 1.61 |  | 1.51 | 1.49 | 1.51 | 1.47 | 1.53 |  | 1.37 |
| Met | 0.48 | 0.52 | 0.50 | 0.48 | 0.47 |  | 0.45 | 0.39 | 0.43 | 0.41 | 0.44 |  | 0.47 | 0.50 | 0.46 | 0.45 | 0.43 |  | 0.44 | 0.46 | 0.44 | 0.43 | 0.46 |  | 0.46 |
| Phe | 0.93 | 1.03 | 0.96 | 0.93 | 0.88 |  | 0.86 | 0.87 | 0.77 | 0.87 | 0.92 |  | 1.01 | 1.04 | 0.97 | 1.00 | 0.87 |  | 1.02 | 0.92 | 0.99 | 1.02 | 0.98 |  | 0.90 |
| Thr | 0.95 | 0.92 | 0.93 | 0.90 | 0.92 |  | 0.85 | 0.82 | 0.85 | 0.81 | 0.84 |  | 1.02 | 0.97 | 0.96 | 0.97 | 0.91 |  | 1.00 | 0.95 | 0.96 | 1.00 | 0.94 |  | 0.97 |
| Try | 0.22 | 0.21 | 0.23 | 0.23 | 0.20 |  | 0.20 | 0.21 | 0.21 | 0.19 | 0.19 |  | 0.23 | 0.24 | 0.22 | 0.25 | 0.22 |  | 0.24 | 0.20 | 0.23 | 0.23 | 0.21 |  | 0.19 |
| Val | 1.03 | 1.18 | 0.98 | 0.93 | 0.93 |  | 0.92 | 0.95 | 0.90 | 0.91 | 0.95 |  | 1.09 | 1.08 | 1.04 | 1.11 | 0.95 |  | 0.99 | 0.90 | 0.96 | 0.98 | 0.95 |  | 0.90 |
| Arg:Lys | 0.44 | 0.62 | 0.85 | 1.01 | 1.16 |  | 0.45 | 0.61 | 0.77 | 1.01 | 1.23 |  | 0.74 | 0.91 | 1.06 | 1.22 | 1.36 |  | 0.87 | 0.97 | 1.21 | 1.39 | 1.50 |  | 0.83 |

^1^ L-arginine was included at 0, 0.3, 0.6, 0.9, and 1.2% in phases I and II to create a total of 5 dietary treatments in each experiment. CJ Bio America INC, Downers Grove, IL. Phases I (d0-d7), Phase II (d7-d21), and Phase III (d21-d43; common diet across Exp. 1 and 2).
